# Acupuncture, or non-directive counselling versus usual care for the treatment of depression: a pilot study

**DOI:** 10.1186/1745-6215-10-3

**Published:** 2009-01-09

**Authors:** Sylvia Schroer, Hugh MacPherson

**Affiliations:** 1 Department of Health Sciences, University of York, York, UK

## Abstract

**Background:**

Depression is one of the most common reasons for consulting in primary care. Acupuncture is a popular complementary therapy choice for depression but its evidence base is poor with more robust high quality trials being required. More than half of depressed patients experience painful symptoms, with severe pain being associated with poor response to antidepressants. Acupuncture may have much to offer as an intervention for depression that also helps alleviate pain. Non-directive counselling is the most widely used psychological approach for depression in NHS settings, and provides a useful pragmatic comparison for acupuncture that would, according to our pre-trial qualitative research, be of high interest to doctors and patients.

**Methods and design:**

The pilot study uses five arms and involves a pragmatic design. All patients will continue to receive usual care. Four groups of patients will be allocated to acupuncture, or non-directive counselling, in addition to usual GP care. The acupuncture and counselling arms will be further split into two groups to explore different treatment regimens. The primary outcome measure is the BDI II. Potentially eligible patients will be screened for depression using the PHQ-9, which is also a secondary outcome measure. Other secondary measures include the SF 36 bodily pain subscale, the CORE OM, the WBQ-12 and the EQ5D. Health economic data will be collected and measures of therapeutic engagement will be used to compare patient's views of therapists and GPs. The study will employ a fully randomised preference design with collection of data on patient preferences and prior expectations.

**Discussion:**

This study has been implemented, and data are currently being analysed to inform the design of a full scale trial. Two practical operational issues that impacted on study implementation are discussed. Firstly, the challenge of recruiting depressed patients via GP consultation. Secondly, the problem of poor uptake and high attrition for counselling and acupuncture, which appeared to be associated with poor questionnaire return, and resulted in missing data. These problems may be relevant to other researchers working in the area of depression, or similar illnesses, where patients may lack motivation and energy to engage in research, or attend for treatment.

**Trial Registration:**

Current Controlled Trials (ISRCTN 59267538)

## Background

### Need for a trial

Depression is one of the most common reasons for consulting in primary care [1]. The economic burden of this health problem in the UK is estimated to be £9 billion per annum with £370 million being due to direct costs of treatment [2]. Despite these considerable costs, current pharmacological and psychological interventions options have limited acceptability and effectiveness. A recent survey of London GPs found an "effectiveness gap" in the treatment of depression, an effectiveness gap being defined as an area of clinical practice in which GPs considered available treatments as not fully effective [3]. Up to 33% of patients do not show an adequate response to pharmacological antidepressant treatment [4], and 30% do not adhere to their medication regime [1]. Patients have expressed the view that there is an over-reliance on prescribed antidepressant medications and that they are keen to have a range of possible treatment choices [5].

A significant number of individuals who are diagnosed and treated for depression also present with painful symptoms, with prevalence estimates ranging from 43% to 65% [6]. A recent survey of 644 respondents conducted by the Depression Alliance found that 99% of people with depression have physical symptoms and 85% believe their "quality of life could be greatly improved if their aches and pains were managed effectively" [7]. GPs are optimally placed to manage these patients with both painful conditions and depression, but feel ill equipped to do so [6]. Patients with multiple painful symptoms and depression are less satisfied with their medical care than patients without mental disorders [8]. Evidence suggests that the presence of pain, particularly if it is severe, may be associated with a poor response to SSRIs [9].

Previous research on the main complementary therapies in the UK has shown that acupuncture is one of the most commonly used CAM (Complementary and Alternative Medicine) modalities in the UK [10], and that depression is regularly treated by acupuncturists [11]. Recent systematic reviews of acupuncture for depression, including one from the Cochrane Collaboration which included seven randomised controlled trials, have concluded that the scientific rigour of studies to date has been too poor to enable robust conclusions to be drawn about the effectiveness of the intervention [12,13]. Reasons for poor methodological quality included: inadequate sample sizes; inadequate reporting; poorly defined outcome criteria; assessors not blinded; and lack of follow-up or longer term outcomes including relapse prevention. Nevertheless, the evidence "generated promising results" [12] and further research is "required" [13]. None of the studies identified by the reviews was conducted in the UK, and in the majority of the trials the acupuncture intervention did not reflect the sort of treatment a person would be likely to receive from a member of the British Acupuncture Council, the leading professional body for acupuncturists in the UK. It is in this context that we set out this proposal for further research to develop a platform for the evaluation of depression, leading to research that will support health care decision makers and patients themselves in making appropriate choices.

### The acupuncture intervention, and selection of a suitable comparator arm

Pre- pilot qualitative research suggested that acupuncture should be provided in addition to usual GP care and that stakeholders (GPs, patients and acupuncture practitioners) would be most interested in a pragmatic trial where the acupuncture intervention would reflect normal clinical practice, and be compared with an existing intervention such as antidepressants, or psychological therapy. Of these choices non-directive counselling was the most practically feasible comparator arm for acupuncture. Non-directive, or 'person centred' counselling as it is also known as, is, according to the CORE database, the approach that is most often used for depressed patients in UK NHS settings in comparison with psychoanalytic or cognitive behavioural methods [14] although it has received considerably less research attention than the latter [15,16]. Non-directive counselling also provides a useful comparison to acupuncture because both therapies involve a similar level of contact time and attention between practitioner or therapist and patient, and a positive therapeutic alliance is thought to be key to both types of intervention. This means the trial can be partially explanatory. However, counselling can only be an approximate time and attention control for acupuncture because both therapies are complex interventions, underpinned by a different theoretical rationale and with expected specific effects.

The acupuncture intervention arm was developed through consulting with over 30 practitioners formally and informally. Quantitative and qualitative research methods were used, the results of which were used to develop a study practitioner's log. This log set out expected treatment parameters and facilitated reporting according to STRICTA guidelines [17].

The counselling intervention was developed through consultation with trial counsellors, who outlined the parameters of the therapy they would provide to trial patients in a written document or 'manual'.

## Study aims and objectives

### Aims

To conduct a pilot for a randomised controlled trial that will evaluate the effectiveness and cost-effectiveness of acupuncture for management of an acute episode of depression and the longer term relapse rates, when provided as adjunct to usual GP care.

To pilot an evaluation of acupuncture versus non-directive counselling that explores pain as a co-variable and investigates other factors that may impact on outcomes, including compliance and acceptability of acupuncture and non directive counselling; personal history of depression; severity of depression; patient preferences; prior expectations; and quantitative indicators of a positive therapeutic relationship (working alliance, enablement and empathy).

### Objectives

In this pilot study our main objectives are:

To estimate the sample size for the full-scale trial based on the variability in the primary outcome measure and likely loss to follow up.

To evaluate recruitment procedures and establish potential recruitment rates.

To explore treatment attendance patterns, potential optimal treatment regimens, and acceptability of treatment protocols to practitioners.

To assess the acceptability and adequacy of trial questionnaires.

To establish a system for monitoring adverse events that is adequate and appropriate.

## Methods

The design is a pilot pragmatic randomised controlled trial of acupuncture for patients in primary care who are suffering from depression with participants being allocated to one of three main intervention arms. Patients allocated to either acupuncture or non directive counselling will be further split into two groups each receiving up to a maximum of either 12 or 24 sessions in order to explore optimal treatment regimens for recovery and relapse prevention. A pilot trial is according to MRC guidelines for the evaluation of complex health care interventions the ideal time to investigate issues such as dosage levels [18].

1. Usual GP care

*2. Usual GP care plus traditional acupuncture*, practised in accordance with Chinese medical theories, and delivered by acupuncturists who are members of the British Acupuncture Council who have been qualified for at least three years. Fifty percent of acupuncture patients will be offered up to 12 sessions to be delivered over a three month period. The remaining fifty percent will be offered 12 sessions in the first three months followed by a further 12 sessions to be delivered over the following/remaining six months of the study.

### Usual care plus non-directive counselling

Patients will be offered counselling in a similar way to acupuncture, i.e. fifty percent will have up to 12 sessions over a three month period and fifty percent will receive up to a further 12 sessions during the remainder of the follow up period (six months).

We will collect data on all treatments provided in all five arms of the trial.

#### Randomisation

Patients will be allocated randomly, by the York Trials Unit, to one of the five groups described above. Researchers and clinicians will have no influence on the allocation of patients.

#### Eligibility

##### Inclusion criteria

Patients who are being managed in primary care who have consulted their GP and have been diagnosed with depression, with a depression score of 10 or above on the PHQ-9 (Patient Health Questionnaire 9) [19], a Diagnostic Statistical Manual IV based screening instrument for depression, and who are over eighteen years of age.

##### Exclusion criteria

Those who have been diagnosed with terminal illness.

Those with mobility issues who cannot travel to appointments.

Those involved with other research projects.

Those with dementia, learning difficulties, and communication problems.

Those currently receiving acupuncture or counselling.

Those who cannot speak sufficient English to communicate with a counsellor or acupuncture practitioner.

Those with alcohol or substance abuse problems.

Those who have received a diagnosis of bipolar disorder, psychosis, or personality disorder.

We will be able to check the patients medical records for all of the above except whether they are receiving acupuncture or counselling, participating in other research and their level of English. We will screen for these in the baseline questionnaire.

#### Outcome measures

##### Primary

BDI (Beck Depression Inventory) [20].

##### Secondary

PHQ-9, CORE 34 [21], SF-36 Bodily Pain [22], EQ5D, and the W-BQ12 (Well-Being Questionnaire) [23]. Outcome measures will be collected by postal questionnaire at three, six, and nine months.

Quantitative process measures at three months will include the PEI (Patient Enablement Instrument) and the CARE (Consultational & Relational Empathy) Measure (CARE) [24,25], the WAI (Working Alliance Inventory-12) [26] as well as measures of satisfaction and adverse events. Participants who are allocated to acupuncturing and counselling interventions will receive additional monitoring in order to evaluate key therapeutic processes (empathy, enablement and working alliance) and assess optimal duration of treatment. The following instruments will be used: the PEI after first and final consultation, and monthly; the CARE measure, after first and final consultation; and the WAI at one month (wording to be modified for acupuncture patients; "therapist" will be substituted with "practitioner" and "therapy" with "treatment"). All adverse events will be continuously monitored by acupuncturists and counsellors, and all adverse events will be collected at three, six and twelve months. Trial questionnaires will collect information about significant life-events at three, six and twelve months. Although such events might be expected to occur randomly a major setback in life might conceivably negate the positive impact of any form of therapy or intervention and should be recorded.

#### Sample size

One of the main objectives of the study is to provide an estimate of the sample size required for the full-scale trial. We plan to recruit forty people into this pilot study. Twelve will receive up to 12 sessions of acupuncture as an adjunct to usual care, with six receiving up to 12 further treatments. Twelve will receive counselling as an adjunct to usual care, with six receiving up to a further 12 sessions. Sixteen will continue to receive usual GP care.

#### Recruitment

We will pilot two methods of recruitment and evaluate related recruitment procedures and rates. Firstly we will use a database recruitment method to recruit those who have already been diagnosed with depression and are still depressed (prevalent cases). These potential participants, who will have received a diagnosis of depression within the previous three years, will be identified from GP databases. Their GPs will send a covering letter informing them about the study, a Participant Information Leaflet, Consent Forms, and Baseline Questionnaire. Based on preliminary searches through a GP database, we estimate that three general practice surgeries in the Selby and York PCT will be sufficient to recruit in full.

Secondly, GPs and mental-health workers within participating practices will refer newly diagnosed (incident) cases of depression, either those with a first episode or a recurrence, into the study. GPs will give eligible individuals a Patient Information Leaflet, Consent Forms and Baseline Questionnaire, and letter inviting them to consider participating in this study.

#### Analysis

Our primary analysis will be to determine the sample size for the full-scale trial. We will conduct an exploratory analysis on an intention-to-treat basis, using analysis of covariance (ANCOVA), which incorporates an adjustment for differences between groups at baseline.

#### Cost-effectiveness

We will pilot an economic evaluation from both an NHS and societal perspective. Data will be collected retrospectively at 3, 6 and 12 months on resource use both within and outside the NHS, including those costs related to all contacts with primary and secondary healthcare services, all contacts with acupuncture practitioners, and counsellors, prescribed and over-the-counter medication, herbs and remedies, private sector health services and any additional complementary therapies. For the pilot, it will be important to determine the key cost drivers, so that we can ensure that this data is accurately collected in the full-scale trial.

#### NHS cost implications

The GP practices will be involved in helping with the database recruitment. This will involve each practice manager in two to three hours work per practice.

#### Trial Management

##### The Trial Team

The trial team includes Dr Hugh MacPherson (Chief Investigator), Sylvia Schroer (Principle Investigator), Dr Joy Adamson, Dr Simon Gilbody, GP Principal Dr David Geddes, and Professor Trevor Sheldon. We will involve users where possible through York & District Mind, Depression Alliance, and/or INVOLVE.

Ethical approval was granted for the study in October 2006 by York Local Ethics Committee with further amendments to questionnaires and information for patients being approved on 4^th ^Jan 2007 (ref: 06/Q1108/56). Originally it was planned to conduct a nested qualitative study to explore patient's beliefs, explanatory models of depression, and expectations of treatment however we decided to pilot quantitative measures instead. The use of these instruments constituted a protocol amendment which required approval.

## Discussion

**Figure 1:**
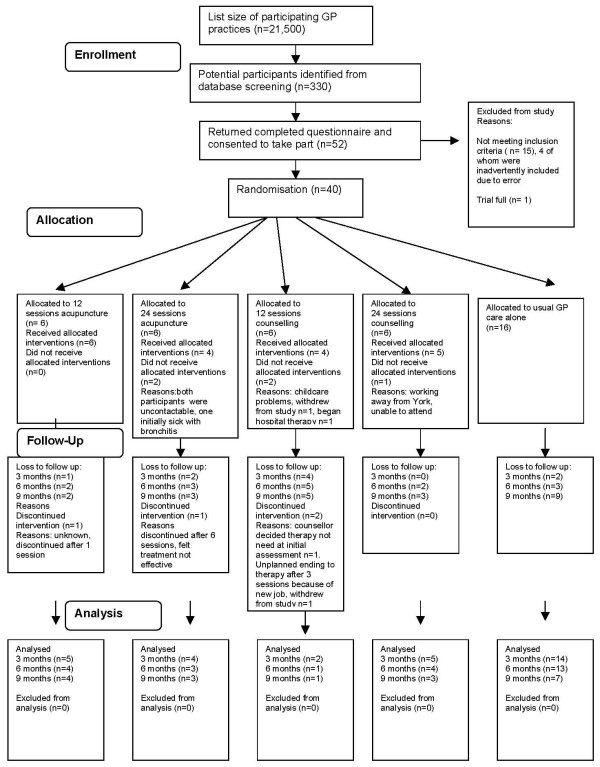
**Study flow diagram**.

Our aim was to conduct a pilot study for a full scale trial of acupuncture for depression, and to look at its potential effectiveness in terms of depression recovery and prevention of future episodes. We sought to investigate the feasibility of comparing acupuncture with non-directive counselling, a widely used primary care intervention, with both therapies being adjuncts to usual GP care. Recruitment began at the end of January 2007. Randomisation took place at the end of February 2007. Data collection was completed at the end of 2007. A CONSORT study flow diagram [27,28] shows recruitment, uptake of interventions and loss to follow up (see Figure 1). Study data are currently being analysed and will be used to inform the design of a full scale trial.

The discussion below focuses on practical and operational concerns involved in performing the study, in particular the problems associated with recruiting depressed patients into trials via their GP, and also concerns about poor uptake and attendance rates for psychological interventions amongst this patient population. These problems may be of interest to other researchers working in the area of depression, or other illnesses, where GPs feel protective of their patients who may also, as a result of their condition, lack motivation and energy to engage in research or have problems complying with interventions.

### The problem of recruiting patients with depression into trials via the GP

GP referrals into depression trials of conventional medical interventions can be problematic. Reasons for poor referral have been investigated by Mason et al, with four themes identified by in depth interviews with 41 GPs. The first was the content and style of depression consultations the second was the wish to protect the doctor patient relationship, the third was the "perceived intrusiveness of introducing research into a complex consultation" and finally the GP's perceptions about their own lack of skill and confidence to introduce research [29]. Our own pre-trial qualitative research suggested that GPs might feel uncomfortable with referring depressed patients to acupuncture because they are not familiar with this intervention in the context of treating depression.

In the event, the use of database recruitment, which circumvented the need for doctors to discuss the trial with patients in a consultation, proved an efficient method. We achieved a recruitment rate of 11% of patients who were invited to participate consenting to take part, who met with the study's inclusion criteria. No patients were recruited by direct referral from any of the fourteen GPs who participated in the trial.

### The problem of 'poor attendance' of acupuncture and counselling interventions

Poor compliance and attendance of mental health services have been identified as a key problem in primary care. For example, a trial of a brief psychological intervention found that non attendance for assessment was 26.1% with only one in five patients referred to the service completing their sessions [30]. Since we were researching a similar patient population, we were concerned about the potential impact of poor uptake and attendance of counselling or acupuncture interventions on the pilot trial. Our resource constraints meant the number of participants in each of the acupuncture and counselling groups amounted to only six. One further problem with poor attendance, or noncompliance, is that intention to treat analysis, the usual method of analysis for randomised controlled trials, is likely to underestimate treatment effects for an intervention, unless noncompliance is negligible [31]. Reasons for poor uptake or attendance of interventions tend not to be random. Attendance patterns for the different interventions can be seen in the study flow diagram. Five out of twenty four participants did not attend any acupuncture or counselling sessions, even when allocated, in two cases (one each for acupuncture and counselling), to the interventions that were believed to potentially be the 'most helpful' for their depression. Eleven out of twenty four participants attended no sessions at all, or less than 50% of their maximum number of allocated sessions. Some participants offered reasons for poor attendance, which included infectious illness, difficulties finding time to attend, problems arranging childcare, and needing to prioritise work commitments. One participant commented that acupuncture had made her feel worse in terms of depression, and prematurely terminated treatment.

Numerous letters were sent to all participants, and phone calls made, to explain the importance of returning questionnaires, regardless of whether interventions had been used or adhered to. Nevertheless questionnaire return in this study was poor: 60% of participants did not return all of the questionnaires for the primary outcome, measured at three, six and nine months, and nearly 80% of participants did not return complete data over the nine month study period. Questionnaire return was especially low amongst poor attenders, and there was a significant association, using Fisher's Exact test, between attendance and questionnaire return for the combined three, six and nine month questionnaires (p value ≤ 0.0001), see Table 1. However, we did not find such an association when we looked at questionnaire return for all of the nine months of questionnaires. This might be attributed to the fact that only 9 out of 40 participants had returned all these questionnaires.

**Table 1:** Attendance and questionnaire return

	**Questionnaire return**			
	**Incomplete for all nine months**	**Complete for all nine months**	**Incomplete for 3, 6, and 9 months**	**Complete for 3, 6, and 9 months**

**Poor Attendance, attended less than 50% of sessions (n = 11)**	11 (35.5%)	0 (0%)	11 (47.8%)	0 (0%)

**Good Attendance, attended 50% or more of sessions (n = 13)**	9 (29.0%)	4 (44.4%)	3 (13%)	10 (58.8%)

**Usual Care**	11 (35.5%)	5 (55.6%)	9 (39.1%)	7 (41.2%)

**Total**	31 (100%)	9 (100%)	23 (100%)	17 (100%)

It may be that we expected too much of our participants, who were after all suffering with depression, an illness that can impact severely on motivation and energy levels. Both acupuncture and counselling can be demanding interventions in terms of the time and effort that is needed to attend sessions. In the full scale trial statistical methods may be used to estimate the clinical effectiveness of interventions, and account for poor attendance or noncompliance [31,32]. This is important if the results of the trial are to address the concerns of patients, doctors, and therapists who are more likely to want to know whether the intervention itself is potentially effective, than allocation to the intervention.

We had believed, on the basis of piloting experience of researching the potential benefits of acupuncture for other health conditions (irritable bowel syndrome, neck pain, and osteoarthritis of the knee), that postal questionnaires would be a suitable method of data collection for this trial. The poor response rates have suggested otherwise and we will need to rethink data collection for the full scale trial. It seems likely that this patient group may need additional support from researchers to help them engage more fully with the research process, particularly if monitoring of trial patients continues for a sufficient period of time to investigate depression relapse as well as recovery from a specific episode.

## Abbreviations

CORE: Clinical Outcomes in Routine Evaluation; GP: General Practitioner; SSRI: Selective Seratonin Re-uptake Inhibitor; NHS: National Health Service; STRICTA: Standards for Reporting Interventions in Clinical Trials of Acupuncture; MRC: Medical Research Council; PCT: Primary Care Trust; CONSORT: Consolidated Standards of Reporting Trials.

## Competing interests

Both SS and HM are acupuncture practitioners and health services researchers. Because of this both authors have made every endeavour to ensure that their research has not been biased in favour of acupuncture.

## Authors' contributions

HM obtained funding for the research project. SS designed the study as part of her ongoing PhD research, with HM and Trevor Sheldon overseeing the design. Her PhD has been supervised by Joy Adamson, Trevor Sheldon, HM and Simon Gilbody. SS wrote the study protocol for submission to ethics, with HM making amendments to it. SS undertook the preparation of all trial materials; recruitment of practices, therapists, patients, and the follow-up of patients enrolled into the study. Data input was organised by HM. Study analysis and interpretation of data is being conducted by SS with the help of Joy Adamson and Mona Kanaan. SS wrote this manuscript. HM and Joy Adamson have made general comments about it.
